# Identification of Bull Semen Microbiome by 16S Sequencing and Possible Relationships with Fertility

**DOI:** 10.3390/microorganisms9122431

**Published:** 2021-11-25

**Authors:** Aleksandar Cojkic, Adnan Niazi, Yongzhi Guo, Triin Hallap, Peeter Padrik, Jane M. Morrell

**Affiliations:** 1 Department of Clinical Sciences, Swedish University of Agricultural Sciences (SLU), 75007 Uppsala, Sweden; yongzhi.guo@slu.se (Y.G.); jane.morrell@slu.se (J.M.M.); 2 SLU-Global Bioinformatics Centre, Department of Animal Breeding and Genetics, Swedish University of Agricultural Sciences, 75007 Uppsala, Sweden; adnan.niazi@slu.se; 3 Estonian University of Life Sciences, 51014 Tartu, Estonia; triin.hallap@emu.ee; 4 Animal Breeders’ Association of Estonia, 79005 Raplamaa, Estonia; peeter.padrik@etky.ee

**Keywords:** bull semen, bacteria, 16S sequencing, sperm quality, fertility

## Abstract

Reports on the use of 16S sequencing for the identification of bacteria in healthy animals are lacking. Bacterial contamination of bull semen can have a negative effect on the sperm quality. The aims of this study were threefold: to identify bacteria in the semen of healthy bulls using 16S sequencing; to investigate the differences in the bacterial community between individual bulls; and to establish if there was a relationship between the bacteria isolated and bull fertility. Semen from 18 bulls of known fertility was used for the DNA extraction and 16S sequencing; 107 bacterial genera were identified. The differences in the amplicon sequence variants (ASVs) and the numbers of genera between bulls were noted. Negative correlations (*p* < 0.05) between several bacterial genera with *Curvibacter, Rikenellaceae RC9-gut-group* and *Dyella* spp. were seen. Other negatively correlated bacteria were *Cutibacterium*, *Ruminococcaceae UCG-005*, *Ruminococcaceae UCG-010* and *Staphylococcus*, all within the top 20 genera. Two genera, *W5053* and *Lawsonella*, were enriched in bulls of low fertility; this is the first time that these bacteria have been reported in bull semen samples. The majority of the bacteria were environmental organisms or were species originating from the mucous membranes of animals and humans. The results of this study indicate that differences in the seminal microbiota of healthy bulls occur and might be correlated with fertility.

## 1. Introduction

One of the main reasons for introducing artificial insemination into animal breeding was to prevent the spread of infectious diseases between males and females [1]. Apart from the urogenital tract itself as a primary source of pathogens [2], there are critical points in semen processing where sperm samples can be contaminated with different types of microbiota [3,4,5]. Therefore, antibiotic use in semen for artificial insemination (AI) is considered to be essential. Different types of antibiotics, singly and in combination, are added to semen worldwide with the aim of decreasing or preventing microbial growth. Antibiotics used in bull semen cryopreservation are mainly based on the regulations given by government directives, e.g., the European Union [6]. However, despite the addition of antibiotics, bacteria can still be isolated from bull semen after thawing [7]. In addition, there is considerable concern about the emergence of antibiotic-resistant bacteria, particularly methicillin-resistant strains [8]. The identification of bacteria in semen samples is a primary step to the rational use of antibiotics and could be helpful in developing the next steps in the control of microbial contamination of bull semen [3]. 

The presence of bacteria in semen does not equate with infection and limited microbial activity does not necessarily affect sperm quality [9]. However, bacteria can have a direct negative affect on sperm quality, depending on the species and number. Negative correlations were reported between certain bacteria and sperm motility [10], viability, membrane integrity and acrosome reaction [11], sperm DNA fragmentation rates [12,13] and the total number of spermatozoa [14,15]. Givens and Marley [16] reported the presence of microorganisms that cause infertility and/or are transmitted via semen. This knowledge can facilitate the identification and exclusion of subclinically infected bulls from production. Bacteria present in the urogenital tract can influence fertility in cows [17]. Postpartum uterine disease caused by pathogenic bacteria not only reduces fertility but also decreases productivity [18]. Postpartum metritis and clinical endometritis caused subfertility in cows by increasing the time to first insemination, delayed conception and increasing the calving to conception interval [19,20]. Furthermore, postpartum clinical endometritis can cause infertility and consequently results in involuntary culling [21]. Marey et al. [22] reported that infections extending from the uterus to the oviduct induce an immune system disbalance that interferes with fertilization. Apart from an effect on the uterus itself, a uterine infection can lead to disruption in the secretion of gonadotropins [23], disruption in ovarian follicle growth and function [24] and a reduction in the oocyte quality [25] (cited from Sheldon and Owens [18]). 

In contrast to these extensive studies in females, the presence and influence of bacteria on sperm quality and fertility in bulls have not been investigated to any great extent. However, the types of bacteria present in semen in various species such as stallions [2,26], boars [27] and rams [28] have been described as well as reports of the influence of bacteria on fertility and sperm quality parameters in humans [29,30]. Interest in the isolation and identification of the main bacteria in bull semen causing infertility began decades ago [31] using culture and identification by means of appearance and biochemical properties; other methods of identification were introduced as the science developed. In general, reports on the use of 16S sequencing for the identification of bacteriospermia in healthy animals are lacking, especially in bulls. The identification of individual opportunistic bacteria is of interest due to the possible transmission between bulls and from bulls to cows, causing economic losses to cattle production. However, there are few studies in which a full microbiological profiling of bull semen has been achieved. 

The aims of the present study were to identify the bacteria in bull semen via 16S rRNA sequencing and to investigate the individual differences in bacterial type and number in bulls at a semen collection station. An additional aim was to examine the relationships between the bacterial community and the overall fertility of the bulls.

## 2. Materials and Methods

### 2.1. Animals and Semen Collection

Ejaculates were obtained from 18 Holstein bulls at an AI center (Animal Breeders’ Association of Estonia, Raplamaa, Estonia) where animals were kept according to national and international regulations. The age of the bulls ranged from 3 to 10 years. The bulls were kept on dry bedding (new sawdust added 4× /24 h) and, if necessary, cleaned by brushing before the semen collection. Semen collection with an artificial vagina is a routine agricultural practice and, therefore, does not require ethical approval according to Estonian law [32]; the bulls at the AI station were not considered to be experimental animals. Therefore, no special ethical permission was required. 

The bulls were prepared for semen collection by allowing them to follow each other in a circular chute for 30–40 min; the bulls were able to make false mounts by jumping on the bull in front but the chute was not wide enough for the bulls to turn round. A sterilized artificial vagina lubricated with Bovivet Gel (Jørgen Kruuse A/S, Langeskov, Denmark) and a sterilized graduated collecting tube were used for the semen collection, which took place approximately 10 m from the laboratory separated by a glass wall. Within a minute after the collection, the sterile graduated tube containing the collected ejaculate was separated from the artificial vagina and passed to the laboratory personnel through a small hatch in the glass wall. All semen collection procedures were performed with sterile equipment and aseptic measures to avoid semen contamination. In the laboratory, an aliquot of 1 mL semen was transferred to an Eppendorf tube above an alcohol burner and stored in liquid nitrogen before transfer to −80 °C for storage until the bacterial DNA extraction was performed. The remainder of the semen samples were processed and used for the evaluation of the sperm motility by CASA. All samples with a total motility >90% and a progressive motility >80% were used for the routine inseminations. The CASA results are presented in Supplementary Table S1. The fertility performance of these bulls was estimated by non-return rates (NRRs) at 90 days post-AI, based on the outcome from the first insemination. In total, 48,469 females were inseminated, comprising 34,800 cows and 13,649 heifers. Miglior et al. [33] defined NRRs as the proportion of cows that are not seen in estrus again after insemination and are, therefore, considered to be potentially pregnant. 

### 2.2. DNA Extraction

The DNA extraction was performed in the Clinical Sciences Research Laboratory at SLU using an AllPrep DNA/RNA/miRNA Universal Kit Cat No./ID 80224 (GIAGE, Germantown, Philadelphia, USA) following the manufacturer´s instructions for the protocol of the simultaneous purification of genomic DNA and total RNA, including miRNA from cells. In total, 10 µL of semen was used for the DNA extraction to reach the maximum amount of 1 × 10^7^ cells, spermatozoa and bacteria according to the protocol. The sperm concentration was evaluated using a Nucleocounter SP100 (ChemoMetec, Allerød, Denmark) according to the manufacturer´s instructions. According to our calculations, 10 µL of semen contained on average 5 × 10^6^ spermatozoa although the bacterial number was not calculated. All samples were centrifuged; the supernatant was removed and only pelleted cells were used. The purity and concentration of the DNA were tested using a NanoDrop 8000 Spectrophotometer (Thermo Scientific, Waltham (HQ), MA, USA). The DNA purity was considered adequate when the 260/280 ratio was between 1.7 and 1.9 and the concentrations were between 5.34 and 10.97 ng/µL. The DNA samples were stored at −80 °C until further preparation.

### 2.3. 16S rRNA Amplification and Sequencing

A two-step amplification protocol was used for the preparation of the V3–V4 16S gene region for Illumina sequencing. The details of the primers used and the cycling protocols are presented in Table 1. The reaction volume of the first step was 15 µL containing 4 ng of the DNA template, 0.25 µM Pro 341F and the same volume of Pro 805R primers with Nextera adaptor sequences (Illumina Inc., CA, USA) as well as 1 µg/µL BSA and 1 × Phusion Taq ready-to-use mix (New England Biolabs, MA, USA). For the second step, the reaction volume was 30 µL and contained 3 µL of the purified DNA template from the first PCR step, 0.20 µM tagged F and R primers with Nextera adaptor sequences and 1 µg/µL BSA and 1 × Phusion Taq ready-to-use mix. Both PCR steps were performed in duplicate and the reactions were pooled and purified between the steps using SeraMag Magnetic Carboxylate Modified particles in a ratio of 1:1. An Agilent Bioanalyzer was used for the quality check. The amplicons were eluted in 10 mM Tris with a pH of 8.5 and stored at −20 °C. The samples were sent for sequencing to SciLifeLab, SNP&SEQ Technology Platform, Uppsala University. Paired-end sequencing was performed on a MiSeq system (Illumina, San Diego, CA, USA) using kit V2. Approximately 40,000 to 60,000 paired-end reads of 250 bp length were obtained for all samples except the control sample (sterile water), which yielded only 13 reads after sequencing.

### 2.4. 16S Profiling

The analysis of the 16S rRNA sequencing data was performed using Nextflow pipeline ampliseq v.1.1.2 (https://github.com/nf-core/ampliseq, accessed on 24 November 2021). Briefly, raw sequencing reads were quality checked initially using FastQC [35] followed by the trimming of the primer sequences from the reads using cutadapt v.2.7 [36]. The raw sequencing data were cleaned from source contamination by running BLAT against the cow reference genome *Bos taurus* 8 available in the UCSC genome browser (https://genome.ucsc.edu/, accessed on 24 November 2021). The sequencing reads were denoised, dereplicated and filtered for chimeric sequences using DADA2 [37]. The denoised paired-end reads were truncated from position 229 (forward) and 215 (reverse) after a manual visualization of the sequencing error profile; all other reads shorter than the cutoff values were dropped. The truncated sequences were merged with at least a 20 bp overlap, resulting in exact amplicon sequence variants (ASVs). These ASVs were taxonomically classified from the phylum to species level clustered with 99% similarity using the SILVA v.132 database [38] by applying a Naive Bayes classifier implemented in QIIME 2 [39] trained on the preprocessed database. Following the taxonomic classification of the ASVs, the ASVs classified as a mitochondria or a chloroplast were removed. Only the ASVs with a minimum read frequency ≥5 in at least one sample were retained for a further analysis. 

### 2.5. Statistical Analysis

The data analysis was performed using R v.3.3.1 software. Pearson correlation coefficients were calculated between the bacterial genera using the cor.test function in the R environment, with *p* < 0.05 being considered significant. The plotting was carried out in R using corrplot v.0.9. A linear discriminant analysis effect size (LEfSe) analysis of the microbial abundance between low and high fertility bulls was performed to detect the differences between the two groups and characterize the biomarkers. The groups were divided based on the NRR where the NRRs were <51% and >51% for low and high fertility bulls, respectively.

## 3. Results

**Figure 1:**
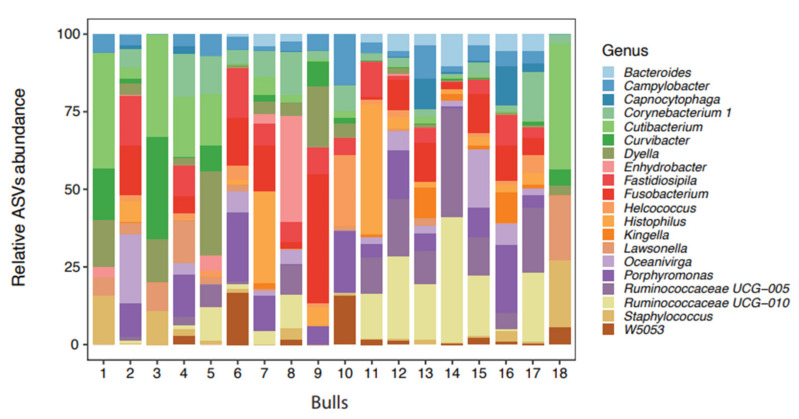
Distribution of the 20 most abundant bacteria genera in the semen from 18 bulls (amplicon sequence variants) identified by 16 rRNA sequencing.

The total amount of DNA in the pool was 1320 ng with a ratio of absorbance at A260/A280 at 1.89 and A260/A230 at 2.1 and a concentration of 24 ng/µL. In total, 107 bacterial genera were identified in 18 bull semen samples. The 20 most frequently seen bacterial genera are presented in Figure 1. 

The bacteria listed among the top 20 for each bull varied considerably; there was also a considerable variation between bulls in both the number of bacterial genera present (ranging from 12 to 89) and in the bacterial count (ranging from 27,827 to 247,273) (Table 2). The highest number of genera identified was in the sample from bull 12, which contained 89 bacterial genera of which 19 came from the 20 most frequently seen. In contrast, Bull 3 was the least colonized, with only 12 bacterial genera present. 

The 20 most frequently seen bacterial genera, starting with the most frequent, were: *Porphyromonas*, *Fusobacterium*, *Ruminococcaceae UCG-010*, *Fastidiosipila*, *Ruminococcaceae UCG-005*, *Cutibacterium*, *Histophilus*, *Oceanivirga*, *Corynebacterium 1*, *Campylobacter*, *W5053*, *Dyella*, *Staphylococcus*, *Lawsonella*, *Helcococcus*, *Bacteroides*, *Capnocytophaga*, *Curvibacter*, *Kingella* and *Enhydrobacter*, which are presented in Figure 1 as the relative ASV abundance.

**Figure 2:**
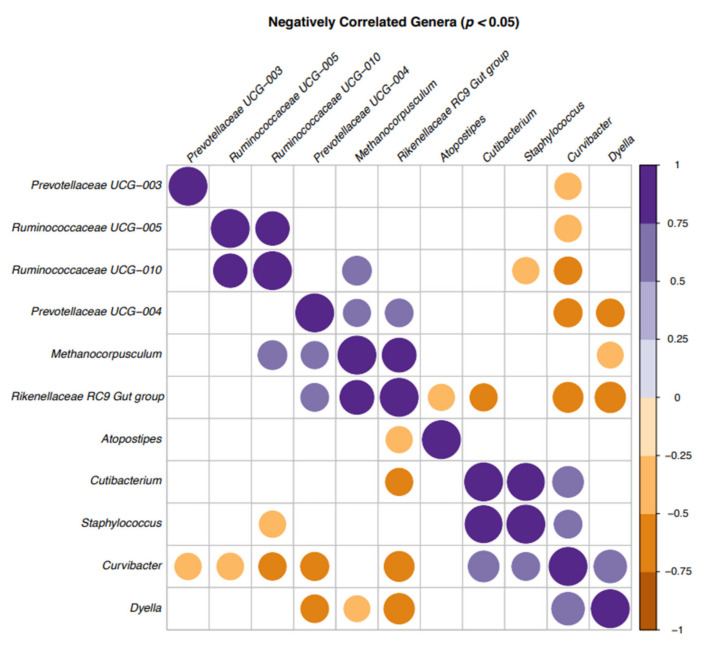
Correlation plot of the bacteria in bull semen identified by 16S sequencing in a correlation matrix showing the significant correlations (*p* < 0.05) between genera: positive (purple) and negative (brown). Blank cells indicate non-significant correlations.

There were negative correlations between several bacterial genera (Figure 2). The majority of the bacteria that showed negative correlations were from the top 20 genera (Figure 1) and included *Curvibacter*, *Cutibacterium*, *Dyella*, *Ruminococcaceae UCG-005*, *Ruminococcaceae UCG-010* and *Staphylococcus* spp. In addition, *Curvibacter*, *Rikenellaceae RC9-gut-group* and *Dyella* spp. (brown spots) were negatively correlated in most cases.

**Figure 3:**
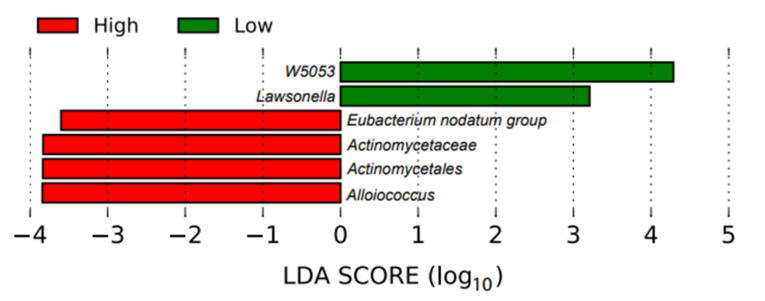
Linear discriminant analysis effect size plot. This plot shows the enriched taxa that were significantly different between the high fertility (red) and low fertility (green) bulls.

In the bar plot (Figure 3), the taxa (including the genus and family) with significant differences between the groups were detected by a LEfSe analysis with a log-10 transformed LDA (linear discriminant analysis) threshold score of 2.0 and a significant *p*-value <0.05. The ASV with a higher LDA score indicated that the ASV was more important according to the LEfSe in discriminating between the low and high individuals. Two genera, *W5053* and *Lawsonella*, were enriched in the low fertility group.

## 4. Discussion

In this study, 16S sequencing of bull semen microbiomes was performed with the aim of identifying the most common bacteria in the semen of healthy bulls. The determination of the most common bacteria genera can enable the development of appropriate control methods.

The isolation and identification of bacteria by commercial microbiological methods are more difficult compared with metagenomics analyses. First, it may not be possible to isolate all the bacteria in a sample. The isolation and identification of bacteria by culture-dependent methods require different culture conditions for different type of bacteria such as the media, temperature, presence or absence of oxygen and time of incubation. The growth of a few bacteria may be inhibited by competition between bacterial species or bacterial overgrowth. Traditional culture-dependent morphological identification methods are time-consuming and do not enable all bacteria to be identified [40]. Although the development of MALDI-TOF (matrix-assisted laser desorption ionization-time of flight) mass spectrometry can help in the process of bacterial identification after culturing, the possibility of identifying isolates depends on the information in the database for the instrument [2]. In contrast, 16S sequencing does not require a bacterial culture and enables the identification of large numbers of bacteria present in a sample [26].

To our knowledge, this is the first comprehensive metagenomic study of the seminal microbiome of healthy bulls. During the introduction of artificial insemination as an animal husbandry technique, there was a need to inhibit bacterial growth in bull semen [41]. Later, culture-dependent studies were conducted to isolate the bacteria responsible for bull infertility [42] and this technique was also used in more recent studies of the microbiological evaluation of frozen semen samples [43]. During the past few years, the identification of individual bacteria from preputial mucosa [44,45] and bull semen [46] was performed with metagenomic methods. Interestingly, there were no significant correlations between the preputial microbial community of bulls of different ages, breed, diet or co-housing [47]. In other species, several studies on culture-independent methods of identification of the normal microbiota in semen have been reported. Stallion semen microbiota was studied by conventional methods of identification as well as MALDI-TOF [2] and recently by 16S sequencing [26]. Al-Kass et al. [26] reported that large numbers of bacterial genera could be identified in stallion semen using metagenomic analyses by 16S sequencing. Based on those studies, variations in bacteria genera and their number between individual stallions and between countries were identified. However, the researchers agreed that the identified bacteria in both stallions and bulls [26,47] were mostly environmental in origin. 

In order to understand the composition of the bull semen bacterial community, it is important to discuss its potential sources. Three of the most common genera identified in our study were *Porphyromonas*, *Fusobacterium* and *Ruminococcaceae UCG-010* respectively. This finding is in agreement with the study of Wickware et al. [47] where *Porphyromonas* and *Fusobacterium* were the most abundant genera in the preputial microbiota of Hereford bulls. These two genera, together with *Bacterioides,* were among the most abundant genera from the upper respiratory and oral mucosal membranes of healthy calves in the first month of life [48,49]. In contrast, the anaerobic bacteria *Porphyromonas*, *Fusobacterium* and *Fastidiosipila* were highly prevalent in most cases of lameness caused by foot lesions [50]. Their presence in semen is probably due to their common occurrence in the environment and subsequent colonization of mucosal membranes.

In the study of Klein-Jöbstl et al. [49] on the microbiota of newborn calves and their mothers, *Ruminococcaceae* was the most abundant type in cow fecal and vaginal samples. In the same study, *Enhydrobacter* was the most dominant in the colostrum on the first day postpartum; this bacterium appeared in our top 20 isolated bacteria, albeit in low numbers. *Ruminococcaceae UCG-005* and *Ruminococcaceae UCG-010*, ranked in the top 10 isolated genera in our study, were also present in the samples of healthy skin in the studies of Bay et al. [50]. Coryneform bacteria *Corynebacterium* and *Cutibacterium* are distributed in the environment in soil and water [51]. These bacteria, as well as *Staphylococci*, are commensals and colonizers of the skin and mucous membranes in animals [52]. *Dyella*, *Helicococcus*, *Capnocytophaga* and *Kingella* are commensal bacteria isolated from the human respiratory tract [53] as well as the oral cavity of humans and animals [54] and are part of the skin flora [55]. Although they are considered to be commensals, all of them were isolated from a patient with severe clinical symptoms; this is the first report of their occurrence in bull semen. Their influence on sperm quality parameters is unknown.

*Histophilosis* is a common disease in North American cattle in the form of septicemia with a high risk of infection and sudden death in calves. A pathogenic form of *Histophila somni* was isolated from the prepuce of a healthy bull and from the vagina of cows with a clinical manifestation of granular vulvovaginitis and abortion [56]. Bovine genital campylobacteriosis caused by *Campylobacter fetus veneralis* or *Campylobacter fetus fetus* is a venereal disease of cattle characterized by infertility, mucopurulent endometritis, early embryonic death and occasionally abortion in systemically healthy cows. As well as venereal transmission, *Campylobacter fetus fetus* can be transmitted by AI in contaminated semen as well as by contaminated instruments. Infections in young bulls can be transient in contrast to older animals with established chronic infections, which may be due to differences in the preputial and penile epithelial surfaces of the lumen and within the crypts in the older animals and the microaerophilic environment that deeper crypts may provide. *Campylobacter* is one of the most demanding bacteria to culture due to its requirement for microaerophilic or anaerobic conditions as well as the need to be cultured immediately after sampling [57]. Therefore, it may be missed when culturing under conventional microbiological conditions. Cagnoli et al. [11] described a significant negative influence of both *Campylobacter fetus veneralis* and *Campylobacter fetus fetus* bacteria species on bull semen quality parameters.

Metagenomic analyses, especially 16S rDNA sequencing, allows the identification of bacteria with unusual phenotypic profiles, rare bacteria, slow growing bacteria and bacteria that cannot be cultured. Furthermore, 16S sequencing can facilitate the definitions of the etiologies of infectious diseases as well as aiding clinicians to choose the most effective antibiotics and determining the duration of the treatment. However, the interpretation of the results can be challenging even for clinical microbiologists [40].

Farahani et al. [58] conducted a systematic review study and meta-analysis of bacteria identified from fertile and infertile men and their influence on sperm quality and fertility parameters. Major differences in the bacterial presence of fertile and infertile men were identified with different sperm quality parameters as well as the negative and positive effects of the individual bacteria on these parameters. Apparent positive effects of *Lactobacillus* spp. on the sperm morphology in addition to a protective effect against *Pseudomonas* and opportunistic pathogens were highlighted. Boud et al. [9] reported that *Pasteurella* spp. abundance was increased in sperm samples with poor motility. Although the latter study showed that the bacterial content might not have an influence on the fertility of men, specific bacterial genera had an impact on sperm morphology and motility. There are few studies on the influence of the bacterial community in semen on sperm quality and fertility outcomes in veterinary medicine. There are a few studies of bacterial influence on sperm quality parameters but usually fertility data are lacking [59]. Cryopreservation does not necessarily reduce the bacterial count. In the study of Reda et al. [60], the bacterial content of cryopreserved semen was evaluated. Their study showed a negative effect of an increased bacterial content on sperm motility, viability and morphology with differences between ejaculates although no differences between the bulls were detected. When boar semen was stored for five days in the presence of an antibiotic, there was an increase in the number of certain bacteria, which was associated with a decrease in sperm motility [61].

In this study, we were interested in the potential associations between the bacteria in the male reproductive tract and the overall fertility of the semen from these bulls. We were not studying the effects of these bacteria on the sperm quality during subsequent storage, which is a different topic. Our results showed that two genera, *W5053* and *Lawsonella*, were enriched in the semen samples from the low fertility bull group. These genera were also present among the 20 most abundant bacteria. Bell at al. [62] described *Lawsonella* spp. as Gram-positive, partially acid-fast, non-spore-forming, anaerobic, catalase-positive and pleomorphic bacteria. Three strains were isolated from human abscesses, which were determined to represent a novel genus (*Lawsonella clevelandensis gen. nov., sp. nov.).* Genus *W5053* is also a novel bacteria; more comprehensive information is lacking. However, a higher abundance of both genera were seen in HIV-infected patients without chronic respiratory diseases [63]. The presence of these two bacterial genera in the semen samples of the bulls with a lower fertility potential is of interest for future research with regard to the origin and potential microbial influence on the sperm quality.

The diversity, number and interaction between the bacteria found in this study put 16S rDNA sequencing high on the list of the methods of choice for the diagnostics of bacterial contamination based on its objectivity and reliability. As most of the bacteria present in bull semen samples originate from the environment or from the mucosa of animals and humans, there is a need for a more effective management of the critical control points during semen collection. However, as 16S sequencing provides information about the presence of bacterial DNA in samples and not specifically about bacterial viability, it can only indicate the likely presence of the bacteria rather than the actual cause of a fertility issue. A possible interaction between the bacteria found in bull semen with a low fertility potential and host bacteria complex interactions would be of interest in future research.

Braga et al. [64] reported that the bacteria of different genera have an influence on microbial community modulation. This microbial coexistence occurs via chemical mediators among bacteria and also between microbes and hosts [65]. Such an interaction may cause alterations in the host physiology [64].

Deines et al. [66] also reported that a host-bacteria interaction, i.e., a host-environment and a bacteria–bacteria interaction, influences the coexistence of microbial species. This study showed that the competitive effect of *Curvibacter* depends on direct contact and indicated that rare microbial community members might be relevant for achieving a native community composition and carrying capacity. Although the genus *Curvibacter* was first mentioned by Ding and Yakota [67], who described three species isolated from well water as the source of origin, there is no previous documentation of its presence in bull semen or other sources. That *Curvibacter* was negatively correlated with other bacterial genera in most cases in this study indicated that this bacterium could be the focus for further research on the influence of the semen microbiota on the fertility of healthy bulls.

The second most negatively correlated bacteria in this study was *Rikenellaceae RC9-gut-group*. It belongs to the *Rikenellacea* family that are recently identified bacteria described as being challenging to culture [68]. This bacterial genus was previously identified in the digestive tract and fecal samples of different animals but not in other types of samples including bull semen. An increased abundance of this bacteria was found in an inflamed human digestive tract but there was no direct indication of their association with disease [69]. The study of Bálingt A et al. [70] showed that *Rikenellaceae RC9-gut-group* with other bacteria increased the sensitivity of the gut to inflammation. Based on the fact that these organisms can be challenging to culture, our current knowledge about these bacteria is based on the information gained from large scale sequencing studies.

The results of the present study indicated that differences in the bacterial microbiota of healthy bulls occur and might be associated with the fertility potential of the bull. Most of the identified bacteria were environmental in origin, indicating that a focus on how bulls are housed and how the semen is processed could help to reduce the bacterial abundance in commercial semen doses. The processing of bull semen should always be performed with a high level of hygiene and microbiological control.

## Figures and Tables

**Table 1:** Primer combination and thermal cycling conditions used to quantify the 16S rRNA.

Primer 16S rRNA	Sequences (5′-3′)	Terminal Cycling	Reference
341F	CCTACGGGAGGCAGCAG	(98 °C 3 min); (98 °C 30 s, 55 °C 30 s, 72 °C 40 s) × 25; 72 °C 10 min; 10 °C hold	[34]
805R	GACTACNVGGGTATCTAATCC	(98 °C 3 min); (98 °C 30 s, 55 °C 30 s, 72 °C 45 s) × 8; 72 °C 5 min; 10 °C hold	

**Table 2:** Number of top 20 and total genera, counts, total counts and non-return rate (NRR, %) per bull.

	Top 20 Genera	Counts	Total Genera	Total Counts	NRR (%)
Bull 1	7	50,127	13	60,678	48.9
Bull 2	20	101,453	69	149,773	48.3
Bull 3	5	17,157	12	56,692	51.4
Bull 4	17	53,773	47	95,193	55.1
Bull 5	12	16,089	31	27,827	61.7
Bull 6	16	188,695	47	247,273	45.9
Bull 7	16	61,305	37	92,055	48.9
Bull 8	17	74,382	49	129,282	52.1
Bull 9	10	53,248	24	70,528	50.4
Bull 10	12	95,853	26	114,379	62.1
Bull 11	18	95,136	82	175,372	51.8
Bull 12	19	70,932	89	130,601	55.2
Bull 13	18	115,553	77	183,004	50.5
Bull 14	18	64,435	76	118,757	47.6
Bull 15	18	102,459	83	170,084	54
Bull 16	19	146,754	53	198,532	52.6
Bull 17	18	68,109	86	141,152	52.3
Bull 18	9	116,226	16	137,258	37

Top 20 genera: 20 most frequently seen genera in all samples; counts: read counts of genera per bull present in the top 20; total genera: number of identified genera per bull; total counts: read counts of genera per bull of all genera per bull; NRR: non-return rate.

## Data Availability

The sequencing data generated in this study were deposited in the European Nucleotide Archive (ENA) under accession number PRJEB47651.

## Supplementary Materials

The following are available online at https://www.mdpi.com/article/10.3390/microorganisms9122431/s1: Table S1: CASA results for the sperm samples from 18 bulls.
