# Supplementary material for: Identification of Bull Semen Microbiome by 16S Sequencing and Possible Relationships with Fertility
Source: Microorganisms. 2021 Nov 25;9(12):2431. doi: 10.3390/microorganisms9122431 (PMC8705814; doi:10.3390/microorganisms9122431)
Supplement: Supplementary file 1 [file microorganisms-09-02431-s001.zip › microorganisms-1479321-supplementary.pdf]

Article

Identification of Bull Semen Microbiome by 16S Sequencing and Possible Relationships with Fertility

Aleksandar Cojkic [1,*], Adnan Niazi [2], Yongzhi Guo [1], Triin Hallap [3], Peeter Padrik [4] and Jane M. Morrell [1]

[1] Department of Clinical Sciences, Swedish University of Agricultural Sciences (SLU), 75007 Uppsala, Sweden; yongzhi.guo@slu.se (Y.G.), jane.morrell@slu.se (J.M.M.)
[2] SLU-Global Bioinformatics Centre, Department of Animal Breeding and Genetics, Swedish University of Agricultural Sciences, Uppsala, Sweden; adnan.niazi@slu.se
[3] Estonian University of Life Sciences, Tartu, Estonia; triin.hallap@emu.ee
[4] Animal Breeders' Association of Estonia, Raplamaa, Estonia; peeter.padrik@etky.ee
* Correspondence: aleksandar.cojkic@slu.se

Supplementary Materials

Supplementary Table S1. CASA results for the sperm samples from 18 bulls.

	Total Motility	Progressive Motility	VCL (µm/s)	VAP (µm/s)	VSL (µm/s)	LIN (%)	STR (%)	WOB (%)	BCF (Hz)	ALH (µm)
Bull 1	91.8	85.8	112.8	71.3	54.7	0.5	0.8	0.6	32.8	3.2
Bull 2	95.1	92.1	118.8	76.1	61.0	0.5	0.9	0.6	33.2	3.2
Bull 3	92.3	90.2	104.0	65.0	47.0	0.5	0.8	0.6	30.7	3.0
Bull 4	97.8	95.9	113.8	74.0	60.9	0.5	0.8	0.7	31.9	3.0
Bull 5	92.4	87.7	97.4	62.5	51.6	0.6	0.8	0.6	32.4	2.6
Bull 6	90.9	84.1	112.8	66.4	50.6	0.5	0.8	0.6	30.5	3.2
Bull 7	90.8	84.0	108.0	66.9	50.4	0.5	0.8	0.6	30.4	3.1
Bull 8	95.8	90.7	100.3	64.8	48.4	0.5	0.8	0.7	30.9	3.0
Bull 9	97.8	93.8	94.1	64.7	50.7	0.5	0.8	0.7	35.3	2.9
Bull 10	97.5	92.4	98.4	66.0	52.1	0.5	0.8	0.7	35.4	2.7
Bull 11	96.7	92.1	117.8	71.9	53.7	0.5	0.7	0.6	30.9	3.5
Bull 12	95.0	88.0	105.0	69.2	59.6	0.5	0.8	0.7	33.9	3.1
Bull 13	95.6	93.4	115.3	74.4	55.7	0.5	0.8	0.6	32.7	3.4
Bull 14	95.6	91.2	112.5	75.3	60.3	0.5	0.9	0.7	33.6	2.9
Bull 15	97.3	92.9	110.3	72.1	54.9	0.5	0.8	0.7	33.6	3.2
Bull 16	91.6	84.9	131.4	81.0	65.2	0.5	0.8	0.6	32.7	3.3
Bull 17	98.8	92.5	112.1	70.4	52.3	0.5	0.7	0.6	31.9	3.4
Bull 18	95.4	90.7	106.5	71.6	56.2	0.5	0.8	0.7	34.2	3.0

CASA - Computer-Aided Sperm Analysis; VCL - Curvilinear velocity (µm/s): the length of the actual path followed by the spermatozoon as a function of time, reported to 1 decimal place; VAP - Average-path velocity (µm/s): the length of the smoothed path followed by the spermatozoon as a function of time, reported to 1 decimal place; VSL - Straight-line velocity (µm/s): the "net space gain" (distance between the first and last track points) as a function of time, reported to 1 decimal place; LIN – Linearity (%): a comparison of the lengths (and therefore relative shapes) of the straight-line and curvilinear paths, reported as an integer percentage (LIN = VSL/VCL × 100; %) ; STR – Straightness (%): a comparison of the lengths (and therefore relative shapes) of the straight-line and average paths, reported as an integer percentage (STR = VSL/VAP × 100; %); WOB - Wobble (%): a comparison of the lengths (and therefore shapes) of the average and curvilinear paths, reported as an integer percentage (WOB = VAP/VCL × 100; %),; BCF - Beat cross frequency (Hz): a de facto expression of the flagellar beat frequency, calculated as the number of times the curvilinear path crosses the average path as a function of time, reported to 1 decimal place; ALH - Amplitude of lateral head displacement (µm): a de facto expression of the

flagellar beat frequency, calculated as the number of times the curvilinear path crosses the average path as a function of time, reported to 1 decimal place.
